# Neodymium acetate as a contrast agent for X-ray phase-contrast tomography

**DOI:** 10.1117/1.JMI.10.5.056001

**Published:** 2023-10-25

**Authors:** Jakob Reichmann, Torben Ruhwedel, Wiebke Möbius, Tim Salditt

**Affiliations:** a Georg-August-University of Göttingen, Göttingen, Germany; b Max Planck Institute for Multidisciplinary Sciences, Göttingen, Germany

**Keywords:** contrast agent, X-ray phase-contrast tomography, neodymium acetate, staining, neuroimaging, retina

## Abstract

**Purpose:**

X-ray phase-contrast tomography (XPCT) is a non-destructive, three-dimensional imaging modality that provides higher contrast in soft tissue than absorption-based CT and allows one to cover the cytoarchitecture from the centi- and millimeter scale down to the nanoscale. To further increase contrast and resolution of XPCT, for example, in view of addressing connectivity issues in the central nervous system (CNS), metal staining is indispensable. However, currently used protocols, for example, based on osmium and/or uranium are less suited for XPCT, due to an excessive β/δ-ratio. In this work, we explore the suitability of different staining agents for XPCT. Particularly, neodymium(III)-acetate (NdAc), which has recently been proposed as a non-toxic, non-radioactive easy to use alternative contrast agent for uranyl acetate (UAc) in electron microscopy, is investigated. Due to its vertical proximity to UAc in the periodic table, similar chemical but better suited optical properties for phase contrast can be expected.

**Approach:**

Differently stained whole eye samples of wild type mouse and tissues of the CNS are embedded into EPON epoxy resin and scanned using synchrotron as well as with laboratory radiation. Phase retrieval is performed on the projection images, followed by tomographic reconstruction, which enables a quantitative analysis based on the reconstructed electron densities. Segmentation techniques and rendering software is used to visualize structures of interest in the sample.

**Results:**

We show that staining neuronal samples with NdAc enhances contrast, in particular for laboratory scans, allowing high-resolution imaging of biological soft tissue in-house. For the example of murine retina, specifically rods and cones as well as the sclera and the Ganglion cell layer seem to be targeted by the stain. A comparison of electron density by the evaluation of histograms allowed to determine quantitative measures to describe the difference between the examined stains.

**Conclusion:**

The results suggest NdAc to be an effective stain for XPCT, with a preferential binding to anionic groups, such as phosphate and carboxyl groups at cell surfaces, targeting certain layers of the retina with a stronger selectivity compared to other staining agents. Due to the advantageous X-ray optical properties, the stain seems particularly well-suited for phase contrast, with a comparably small number density and an overall superior image quality at laboratory sources.

## 1 Introduction

Microscopy—both in its two-dimensional (2D) or three-dimensional (3D) variants—requires generation and variation of contrast, often enabled by chemical staining or labeling. Classical histology and histo-pathology of thin tissue sections relies on a portfolio of established staining agents, such as H&E (hematoxylin and eosin) to name the most common example. Suitable stains exhibit preferential binding to different cellular organelles and biomolecular constituents, such as nuclei, extracellular matrix, and cytoplasm. Specific antibody labels create exclusive contrast for desired target proteins. Highest sensitivity down to single molecule level is enabled by optical fluorescence techniques, including the super-resolution microscopy techniques, such as stimulated emission depletion microscopy (STED), photo-activated localization microscopy (PALM) or stochastic optical reconstruction microscopy (STORM)^1^ for which specific dyes were developed.^2^ Larger penetration depth and field of view (FOV) in particular for the investigation of tissues are offered by light-sheet microscopy,^3^ again relying on stains or specific labels. Electron microscopy (EM) of biological tissue also takes advantage of well established heavy metal stains, notably by the classical protocols based on osmium tetroxide (${\mathrm{OsO}}_{4}$), uranyl acetate (${\mathrm{UO}}_{2}{(\mathrm{CH}}_{3}{\mathrm{COO})}_{2}$), or lead citrate $C_{12}H_{10}O_{14}{\mathrm{Pb}}_{3}$. More recent developments, such as rOTO,^4^ are instrumental for labeling brain tissue^4^^,^^5^ in view of connectivity studies. This has, for example, enabled high resolution reconstruction of neuronal networks of mouse retina.^6^ 3D tissue imaging by EM is enabled by serial block-face electron microscopy or focused ion beam scanning electron microscopy but is still very limited in volume throughput, and depending on volume size can take several days to weeks, strongly restricting the feasibility of high-throughput comparative studies. Sample preparation and staining pre- or post-sectioning can take days and require a lot of experience and manual processing of the sample.

Phase-contrast X-ray computed tomography (XPCT) based on image formation by free space propagation complements optical and EM techniques. It covers an intermediate resolution range from several microns down to the range of 10 nm and offers high bulk penetration and volume throughput. In addition, XPCT can be implemented in multi-scale approach, covering larger tissue scales up to the entire organ level. XPCT has even been implemented for *in-vivo* imaging of free breathing mice^7^ and can quantify morphology and cytoarchitecture of biological tissue.^8^^–^^10^ Different pathologies of the central nervous system (CNS), such as, e.g., Alzheimer^11^ or multiple sclerosis,^12^ have been addressed by virtual histology based on XPCT. The acquired volumes can be analyzed by advanced statistical methods, for example, based on optical transport theory,^13^ machine learning algorithms,^14^ or shape measure analysis.^9^^,^^15^ Importantly, XPCT has been introduced for unstained tissues, including formaldehyde-fixed paraffin embedded tissue blocks, fully compatible with clinical pathology.^8^^,^^16^

Notwithstanding the benefits of unstained preparations and 3D imaging of electron density without additives, it is evident that contrast variation based on stains or labels is an asset that should also be exploited in XPCT. In this way, structural features that are visible in synchrotron radiation (SR) can be contrasted also in instruments with much lower partial coherence,^17^^,^^18^ or individual neurons can be contrasted with respect to the tissue background, for example, by the Golgi-Cox staining protocol.^19^ To this end, radiocontrast agents are used,^20^ which increase X-ray attenuation by accumulating heavy atoms with high atomic number Z at different tissue sites. Well known examples are barium-, lead-, or iodine-based agents, phosphotungstic acid, and gold nanoparticles to mention a few. Radiocontrast agents are rarely optimized for XPCT but are typically translated either from clinical radiology and hence optimized for absorption contrast or from EM, again strongly inclined toward absorption. Instead, one would seek a balance between increase of phase shift while keeping absorption tolerable, depending on the specific application. Since phase shift is proportional to the projected electron density, a heavy atom contrast agent is both an enhancer for attenuation as well as for the phase signal. In terms of atomic form factors $Z+f^{\prime }(E)+if^{\prime \prime }(E)$ describing the atomic interaction with X-rays of photon energy E, for a stain optimized for phase instead of absorption contrast, one would prefer atomic labels, which are located in the upper right corner of a plane spanned by the axis $Z+f^{\prime }$ and $(Z+f^{\prime })/f^{\prime \prime }$. This is because labels with high $Z+f^{\prime }$ values warrant high contrast, which is desired, but at the price of poor phase retrieval if $f^{\prime \prime }$ becomes too high. Note that for given concentrations, this choice of atomic form factors directly translates to high δ and high δ/β ratio. For the typical photon energy range 5 to 50 keV where XPCT by free space propagation is performed, one may search within a surprisingly large range 24≤Z≤64, in order to be well above the corresponding L-edges and below the K-edge (in order to keep $f^{\prime \prime }$ reasonable). This would exclude commonly used radiocontrast agents W, Os, Pb, or U, at least for bulk labeling. Note that iodine based stains^21^ as well as recently developed *Br* based eosin stains for the cellular cytoplasm^22^ meet this criterion, whereas the Pb based hematein stain targeting cellular nuclei^23^ would hence not fall into this category.

Interestingly, several stains that have recently been proposed in EM as replacements of commonly used highly toxic or radioactive labels, such as osmium tetroxide (${\mathrm{OsO}}_{4}$) or uranyl acetate (UAc), may be interesting candidates for XPCT. Notably, a few years ago UranyLess was developed as a safe and non-radioactive stain while providing many of the other characteristics of UAc.^24^ Most recently, neodymium acetate (NdAc) was proposed as another substitute and was used to replace UAc in several routine applications.^25^ Belonging to the same side group in the periodic table, oxidation states and chemical properties are similar, but at the same time a higher $(Z+f^{\prime })/f^{\prime \prime }$ ratio compared to UAc is reached. Note that for the same number density of atoms, this equally corresponds to an increase in the δ/β ratio, where n=1−δ+iβ denotes the X-ray index of refraction.

Here, we study the suitability of NdAc for XPCT. Intact murine eyes as well as other parts of the CNS were prepared and stained with NdAc as well as alternative stains. We compare the imaging results qualitatively and quantitatively and evaluate the suitability of NdAc for high resolution XPCT. Inline with the ubiquitous assumption of a homogeneous object, we reconstruct the XPCT data based on a fixed coupling of β and δ. This parameter is selected by varying this parameter in the reconstruction, around the calculated values (for the assumed elemental composition).

Murine eyes were selected as test samples since they allow easy penetration of the stains and consist of multiple tissue layers as well as the retina, with all structures showing different chemical compositions and therefore different binding affinities. In previous studies, mammalian and insect eyes were proven to be suitable objects to be investigated by XPCT.^26^^,^^27^ Also, there are indications that neurodegenerative diseases are preceded by degeneration of the retinal structure, making it an important object to study the origins and progression of conditions, such as Parkinson’s disease^28^ or Alzheimer’s.^29^ The retina consists of the following layers (optical nerve side to eyeball): choroid, retinal pigmented epithelium, layer of rods and cones, outer nuclear layer, outer plexiform layer, inner nuclear layer, inner plexiform layer, Ganglion cell layer, nerve fiber layer, and the internal limiting membrane where the incident light falls in.

Hence, the main goal of the this paper is to investigate the potential benefit of using NdAc as a contrast agent in XPCT by evaluating its optical properties and by comparing it to ${\mathrm{OsO}}_{4}$ and iodine stained murine eyes samples. The paper starts with the description of the staining and embedding procedure (Sec. 2.1) and is complemented with a brief outline of the tomography setups, namely the commercial laboratory instrument equipped with a nano-focus source (Sec. 2.2), and the custom built synchrotron holo-tomography endstation GINIX at beamline P10 of PETRA III storage ring (DESY, Hamburg) in Sec. 2.3. The results achieved with the two setups are subsequently presented in Secs. 3.1 and 3.2. Finally, the staining results are quantified and evaluated in Sec. 3.3, tomographic reconstructions are visualized by 3D renderings. The work closes with a discussion of the results and a brief outlook in Sec. 4.

## 2 Methods

### 2.1 Staining and Embedding Procedure

Murine CNS tissues from previously dissected laboratory mice (75d old, C57N/Bl6 mice, sacrificed for a different biomedical research project) were used to showcase and compare the staining agents of interest here. The effective uptake of ${\mathrm{OsO}}_{4}$ by various retinal layers was already demonstrated in a preceding high resolution XPCT study.^30^ Besides fully intact eyes, tissue from murine cerebellum and corpus callosum was collected to investigate the binding behavior of the different staining agents, namely ${\mathrm{OsO}}_{4}$, Lugol’s iodine solution (LIS), and NdAc.

The mice were perfused or immersion-fixed with a K+S solution. After several washing steps in 0.1 M phosphate buffer (PB) (3×15  min), the tissue (diameter∼1.5  mm) was post-fixated with 2% ${\mathrm{OsO}}_{4}$ at 4°C (4 h). To this end, 4% ${\mathrm{OsO}}_{4}$ stock solution was mixed in a 1:1 ratio with 0.2M PB. In the next step, the tissue was repeatedly washed (3×15  min) in the same buffer and dehydrated with increasing concentration of acetone (1 h) and embedded by infiltration with 2:1 acetone/EPON (1.5 h), 1:1 acetone (1.5 h), 1:2 acetone/EPON (overnight), and pure EPON (4 h).^31^ Finally, the samples were mounted in polyimide (Kapton) tubes and polymerized for 24 h at 60°C. Staining with ${\mathrm{OsO}}_{4}$, LIS, and NdAc was conducted in the same way. The staining protocols for ${\mathrm{OsO}}_{4}$ and LIS are already well established in X-ray tomography and proved to enhance contrast by binding to different regions of biological tissue.^21^^,^^32^

**Fig. 1:**
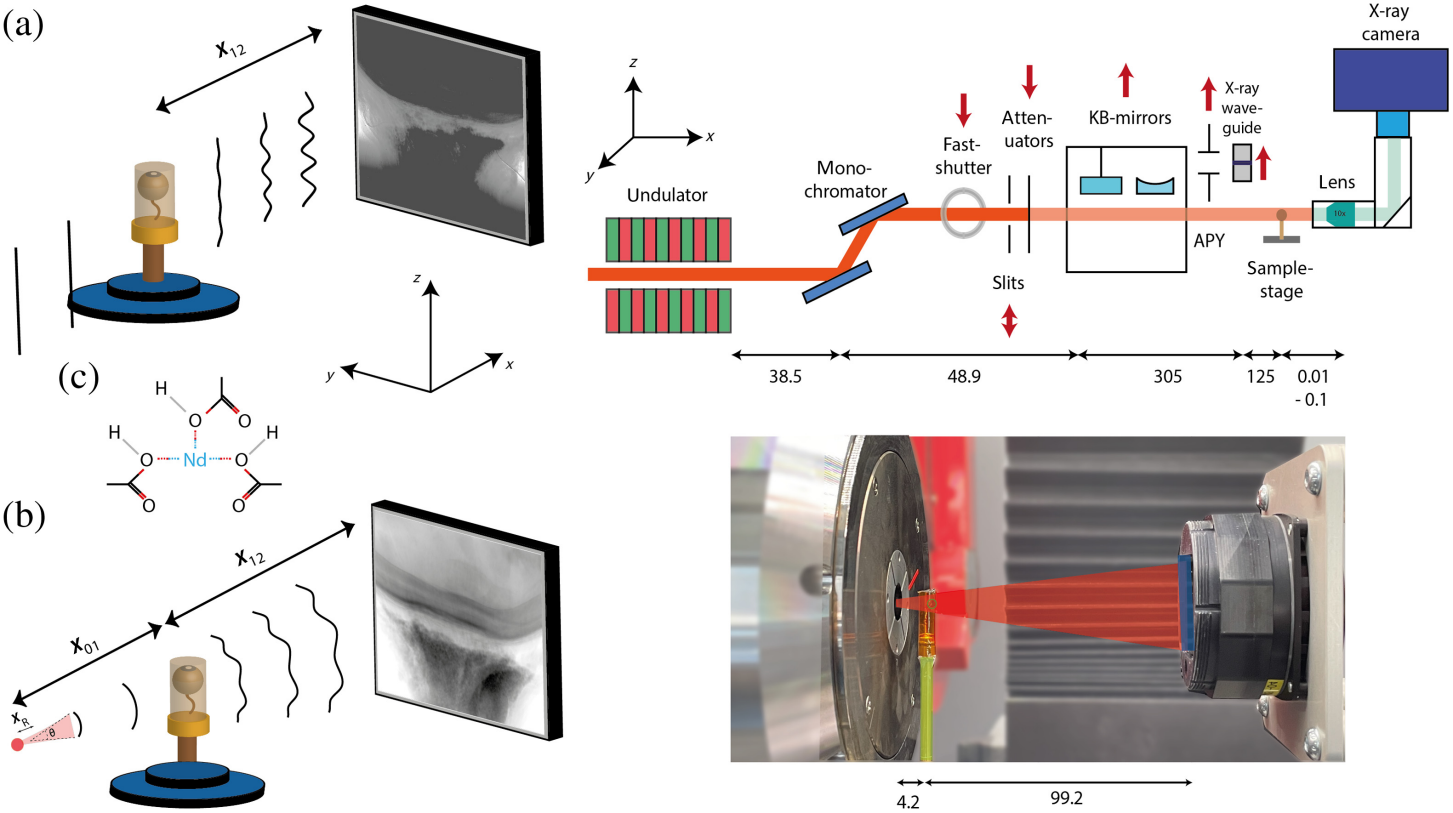
(a) Schematic overview of the parallel beam setup at the GINIX endstation with an attenuator in and KB-mirrors and X-ray waveguide out of the beam path (right). The propagated exit wave of a given sample projection impinges on the detector at a distance $x_{12}$ behind the sample, chosen to reach the holographic regime (left). (b) Laboratory nano-CT imaging setup with opening angle θ, a spatial coherence length $\xi_{⊥}$. The ratio of source-to-sample ($x_{01}$) and sample-to-detector ($x_{12}$) distance (left) defines the magnification $\left[(x_{01}+x_{12})/x_{01}\right]$. Photo of laboratory setup (right): sample holder (green), beam (red), and detector area (blue). (c) 2D chemical structure of NdAc. A trivalent cation is coordinated by three acetate molecules that are formed by combination of acetic acid with a base. All lengths are in mm.

Since Nd and U belong to the same side group of the periodic table, similar chemical properties with regard to binding and clustering at different regions is expected and can indeed be observed, see for example Fig. 1(c). In buffer, Nd is present as a trivalent cation that accumulates preferentially at anionic groups, in particular phosphate groups in nucleic acids, phosphate, and carboxyl groups, such as gangliosides, mostly present on cell surfaces.^33^ NdAc is applied in aqueous solution (4%) to give sufficient contrast, which could be further enhanced by a higher NdAc concentration or, e.g., a replacement of water by a saturated methanolic solution.^34^ In contrast to UAc, NdAc is neither radioactive nor highly toxic, significantly facilitating the handling and staining procedure.

### 2.2 Laboratory Nano-CT Imaging

While synchrotron imaging is still regarded as the gold standard in XPCT, state-of-the-art laboratory sources can provide sufficient flux and coherence for in-house (phase-contrast) tomography with voxel sizes in the range of a few microns down to the sub-micron range. Recent results have opened up a promising approach to study biological tissues in the laboratory^18^^,^^35^^,^^36^ and to translate 3D virtual histology from synchrotron to compact laboratory sources, important for pre-clinical and clinical research.

While the tissue samples have been scanned at several different custom built in-house CT instruments, we here present results obtained at the commercially available EasyTom nano-CT system (RX Solutions, France). The system allows for switching detectors and sources and flexible adjustment of geometry and source parameters well suited to optimize the setting for a specific application. In the present case, the nano-CT source was used with a LaB6 cathode and a transmission anode with an adjustable spot size (0.4 to 1.5  μm), a CCD camera (Gadox scintillator with free fiber optics coupling, 9  μm pixel pitch, and 2016×1344  pixel after 2×2 binning), and a custom-made sample holder. High geometric magnification was selected by moving the sample close to the source spot Fig. 1(b). $N_{\mathrm{prj}}=1568$ images were recorded over 360 deg with a tube voltage of 80 keV and a polychromatic spectrum. Accumulation of several images at the same position was used to improve the signal-to-noise ratio (SNR). Before the scan, the reference images at selected angles were recorded for subsequent tracking and correction of spot size movement over the duration of the scan. Flat field images were recorded inside the EPON material to receive normalized intensities in the projection images by correction for low-frequency effects.^37^ The exact settings are given in Table 1.

**Table 1:** Parameters of tomographic scans for stain comparison at laboratory source.

	Unstained eye	${\mathrm{OsO}}_{4}$ eye (1 h)	NdAc eye	NdAc CC	NdAc cerebellum
Energy (keV)	80	80	80	80	80
Detector type	CCD	CCD	CCD	CCD	CCD
Exposure time (s)	2.5	2.5	2.5	2	2
# Acc	12	12	12	6	6
FOV (px)	1983×1224	1983×1224	1983×1224	1983×1226	1986×1231
Voxel size (μm)	0.73	0.73	0.73	0.79	2
$x_{01}$ (mm)	4.2	4.2	4.2	4.5	12.1
$x_{02}$ (mm)	103.4	103.4	103.4	103.4	108.4

After the scan was completed, the reference images were used to account for source spot movement, and the rotation axis was corrected. Finally, ring filter correction and phase retrieval algorithm were applied before the actual tomographic reconstruction. For phase reconstruction, a Paganin-based phase retrieval algorithm was used and the according parameter (δ/β) was set manually.^38^ Fiji^39^ and MATLAB (R2020a) were used for further analysis and NVIDIA IndeX^40^ for rendering of the volumes. Video 1 shows an entire rendered optical nerve with retina scanned at the laboratory setup (Avizo, Thermo Fisher Scientific, Germany).

### 2.3 Synchrotron-Based X-Ray Tomography

SR sources provide users with highly brilliant and coherent X-ray beams that allow one to exploit quantitative phase contrast on multiple scales and correspondingly different geometries. In this experiment, the parallel beam tomography setup^41^ of the GINIX endstation^42^ at the P10 beamline of the PETRA III storage ring (DESY, Hamburg) was used. Due to the high number of photons, a continuous rotation and short exposure time is sufficient to fill the dynamic range of a 16-bit detector, resulting in reduced moving artifacts and a short overall scan time (≈2  min). For the present measurements, single-distance tomograms with 3000 projections over 360 deg were recorded. The monochromaticity (Si(111) channel-cut monochromator) allows the tissue to be studied at a well defined photon energy instead of the broad bandpass unavoidable for in-house CT. The sample’s optical indices and imaging parameters fall within the regime where CTF-based phase retrieval is suitable to solve the phase problem.^43^

For the setup, the sCMOS camera pco.edge 5.5 (PCO, Germany) with a 100 Hz maximum frame rate, 2560 × 2160 pixel, a rolling shutter, and a fast scan mode was mounted on an high resolution detection system (Optique Peter, France) with a 50 mm-thick LuAG:Ce scintillator and a 10× magnifying microscope objective.^41^ This resulted in an effective pixel size of 0.65  μm and a total exposure time of about 75 s. Focusing optics such as KB-mirrors or waveguides as well as the fastshutter were moved out of the beam path, whereas the beam size was adjusted by an upstream slit system resulting in a beam size of about 2  mm×2  mm [Fig. 1(a)].

With a sample-to-detector distance (SDD) of 149 mm, the holographic regime was reached with a Fresnel number of

$$F=\frac{px_{\mathrm{eff}}^{2}}{x_{12}\lambda }\approx 0.0366,$$ (1)

for E=16  keV, wavelength λ, and an effective pixel size $px_{\mathrm{eff}}$ of 0.65  μm. Phase reconstruction was carried out using the linearized single step CTF-approach,^43^^,^^44^ which is implemented in our code package HoloTomoToolbox.^45^

Tomographic reconstruction was carried out after flat- and dark field correction with the *iradon* function implemented in MATLAB using a Ram-Lak filter for backprojection. To avoid ring artifacts, hot pixel in the projections was removed by replacing them with the mean of adjacent pixel. The Fresnel number was verified and if needed refined by inspecting the radially averaged PSF of a hologram to the expected CTF and according re-scaling. In addition, a wavelet-based filtering approach was applied to further mitigate ring artifacts occurring during the reconstruction.^46^ For post processing and visualization, the Fiji software was used.

## 3 Results

### 3.1 Laboratory Comparison

#### 3.1.1 Murine eye imaging

**Fig. 2:**
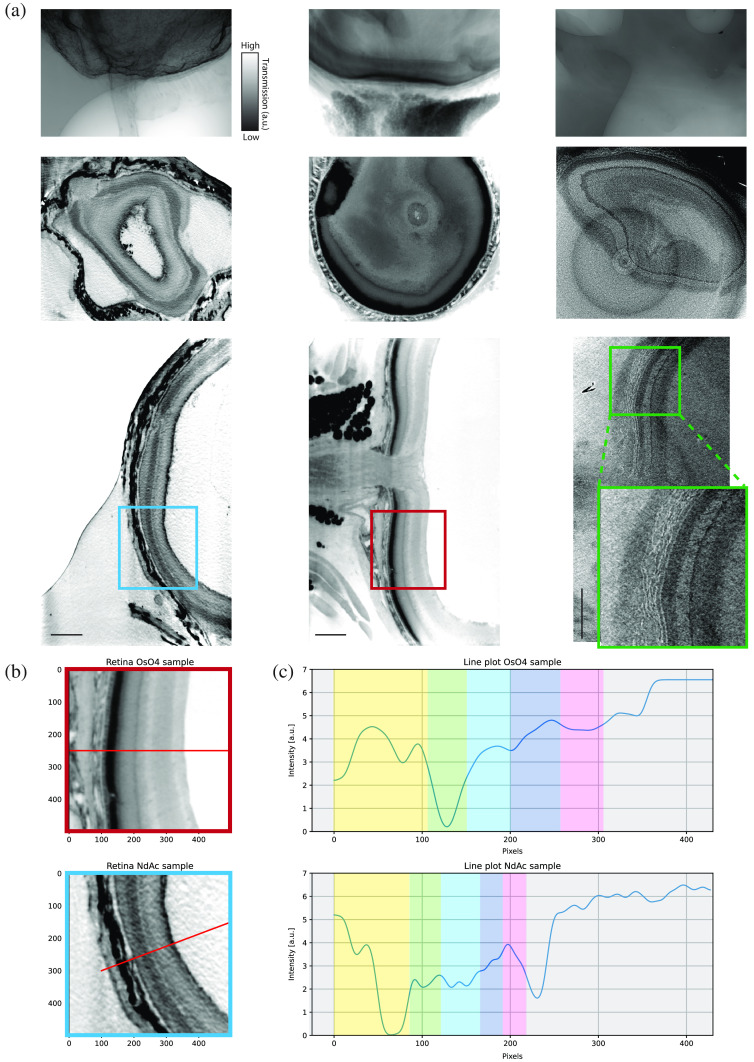
Comparison between NdAc (left), ${\mathrm{OsO}}_{4}$ (center), and unstained (right) entire murine eye samples by in-house nano-CT system. (a) First row shows a projection image of each dataset. The second row depicts a selected slice from the xy-plane (maximum intensity projections over five slices) and the third row from the yz-plane. In (b) and (c), smoothed line profiles from a similar region in the ${\mathrm{OsO}}_{4}$ and NdAc sample are plotted. Each colored region represents a layer of the murine eye. Yellow, sclera; green, choroid; light blue, rods and cones layer; dark blue, nuclear/plexiform layers; pink, ganglion cell layer. Scan settings and reconstruction parameters are given in Table 1. Scale bars: 200  μm.

We first present in-house scans of EPON embedded murine eyes, stained either with NdAc or ${\mathrm{OsO}}_{4}$ as well as an unstained reference sample, see Fig. 2(a). Already based on visual inspection of the projection images, which were recorded with the exact same settings (Table 1), clear differences between the samples can be observed. While the unstained tissue shows a general lack of contrast, which is reflected by a very low SNR in the reconstructed images, different regions of the retina are contrasted and emphasized in the stained preparations. The projections of the NdAc treated retina show mainly the surfaces/interfaces of the tissue to be contrasted, whereas ${\mathrm{OsO}}_{4}$ seems to reach a higher and more bulk-like penetration and binding, resulting in a more uniform and stronger attenuation in all regions. A qualitative analysis of the intensity profiles across the retinal layers in ${\mathrm{OsO}}_{4}$ and NdAc stained layers indicates larger deviations of attenuation values depending on the according layer, thus suggesting a higher specificity of the NdAc stain. In addition, different retinal layers show higher absorption properties. While, e.g., in the ${\mathrm{OsO}}_{4}$ stained sample mostly the choroid layer is strongly targeted, the NdAc shows a high binding affinity to the sclera, the internal limiting membrane as well as rods and cones in the retina. In addition, cells in the Ganglion cell layer are highlighted and their distribution can be investigated. We also note that the NdAc stained region seems to be slightly compressed in comparison to the ${\mathrm{OsO}}_{4}$ stained sample (≈60μm thinner), which might be attributed to the selection of the compared slices as well as to sample preparation artifacts or differences in specimens.

#### 3.1.2 Murine brain imaging

**Fig. 3:**
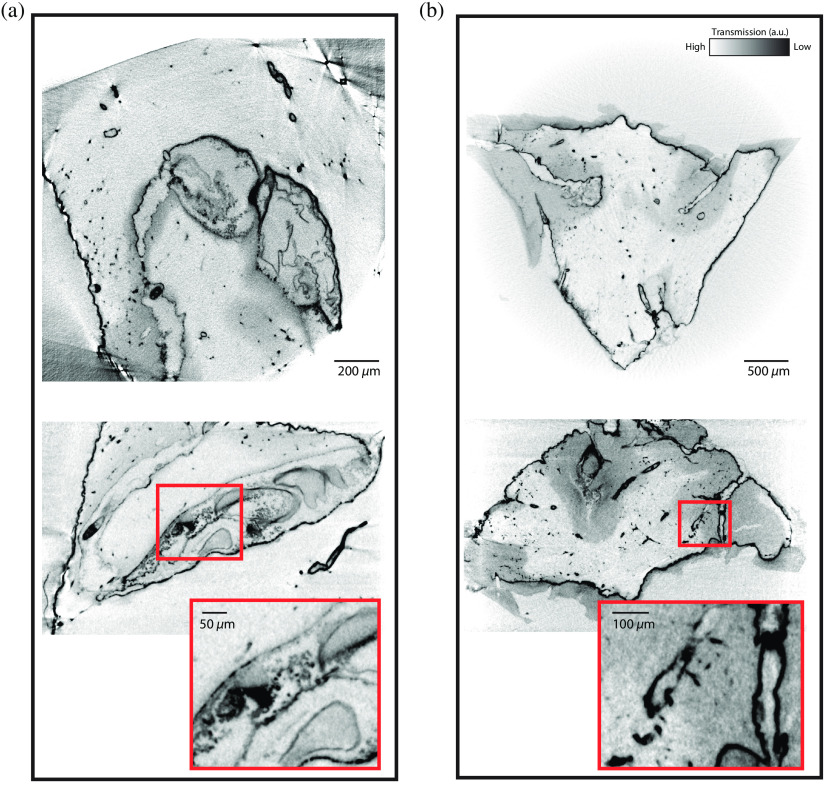
(a) Corpus callosum and (b) cerebellum stained with NdAc shown in xy-plane (top) and xz-plane (bottom), both as maximum intensity projections over five slices, recorded with the laboratory setup. Scan settings and reconstruction parameters are listed in Table 1.

Murine corpus callosum and cerebellum were also imaged in the laboratory setup to investigate the binding behavior of NdAc in different neuronal tissues (Fig. 3; Table 1). It can be observed that in both cases, surfaces and interfaces are pronounced, possibly because these interfaces are defined by well exposed cell surfaces with anionic phospholipids. Also, vasculature seems to be affected by the stain and the whole vascular network becomes easily distinguishable from the surrounding tissue, which opens up a promising approach for rendering and segmentation of the vascular tree. In the cerebellum, gray matter is also clearly separable from white matter. Cell layers on the other hand could not be clearly be resolved. This could be explained based on insufficient sampling and resolution, or—more likely—by a lack of contrast, which could either result from a low penetration depth of the stain, the compactness, and higher tissue density, or to the lower binding of Nd in these regions.

### 3.2 Qualitative Comparison of Different Staining Agents Using Synchrotron Phase-Contrast CT

**Fig. 4:**
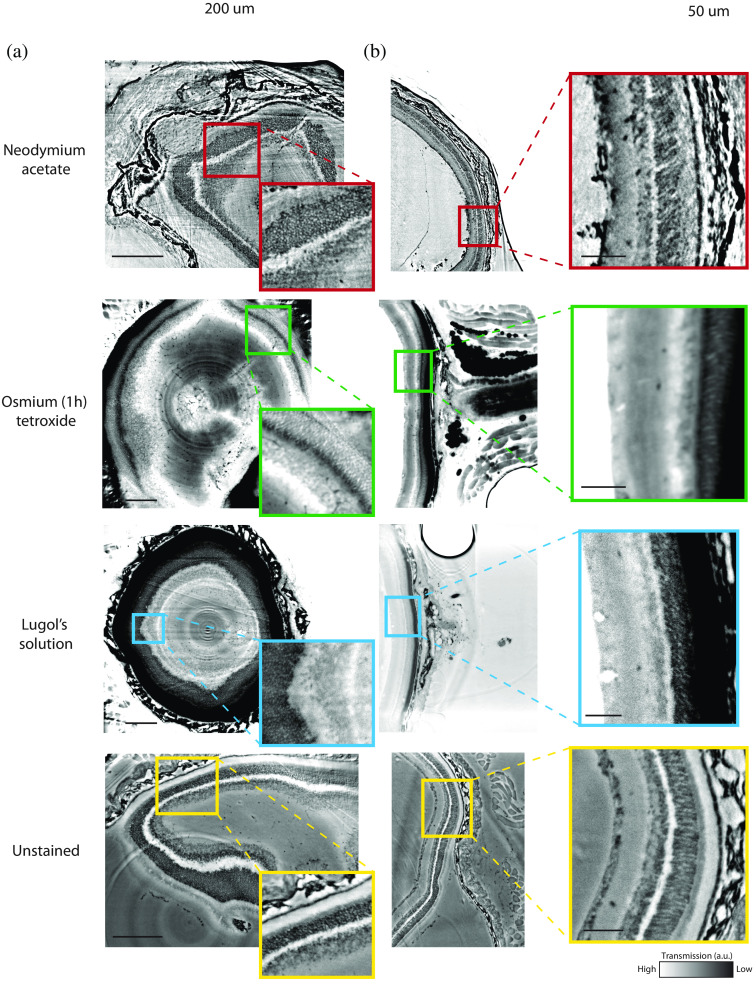
Comparison between NdAc, ${\mathrm{OsO}}_{4}$, LIS stained and unstained entire murine eye samples scanned in parallel beam geometry at 16 keV at the synchrotron facility (SDD: 149 mm). (a) A selected slice from the *xy*-plane (scale bar: 200  μm) and (b) a slice from the yz-plane with a zoomed section (scale bar: 50  μm). Scan settings and reconstruction parameters are given in Table 2.

Synchrotron data were collected from a NdAc, ${\mathrm{OsO}}_{4}$, Lugol’s solution stained retina, and compared with an unstained sample at 13.8, 16, and 20 keV. In Fig. 4, reconstructed slices are presented for all samples scanned at 16 keV since this photon energy yielded the most comparable results.

**Table 2:** Parameters of synchrotron measurements of different stains with a parallel beam setup.

	Unstained	${\mathrm{OsO}}_{4}$ (1h)	Lugol	NdAc
Energy (keV)	13.8 and 16	16 and 20	13.8 and 16	13.8 and 16 and 20
Exposure time (ms)	35	35	35	35
FOV (mm)	1.6×1.4	1.6×1.4	1.6×1.4	1.6×1.4
Voxel size (nm)	650	650	650	650
SDD (mm)	29 and 149	29 and 149	29 and 149	29 and 149
Phase retrieval	CTF	CTF	CTF	CTF
δ/β (@ 16 keV)	35	10	35	5

**Fig. 5:**
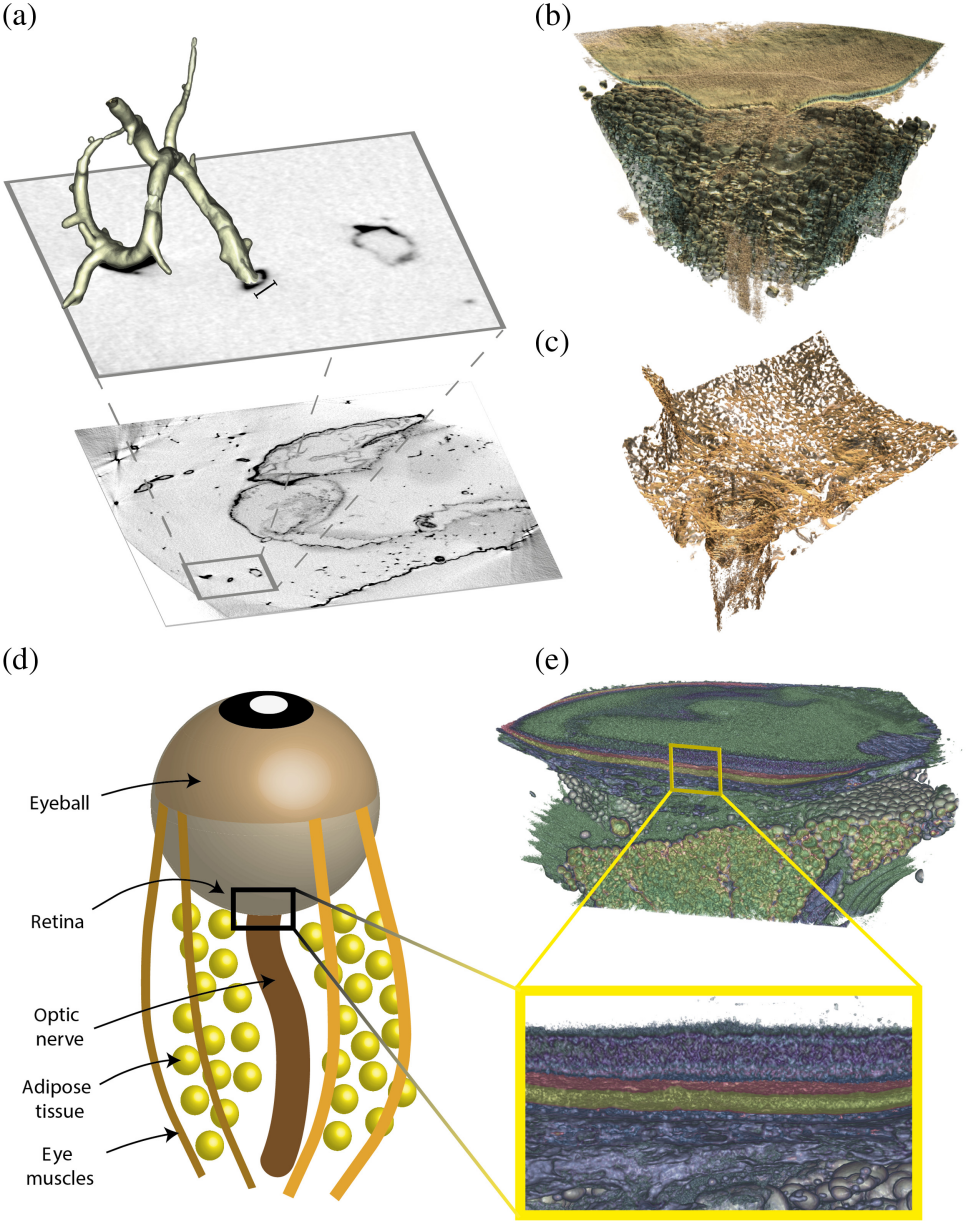
Rendering with NVIDIA IndeX. (a) A part of a vascular tree extracted from a NdAc stained murine corpus callosum [see Fig. 3(a)]. Scale bar: 40  μm. (b) An ${\mathrm{OsO}}_{4}$ stained rendered eye sample, where the adipose tissue and eye muscles are clearly visible (green), whereas the optical nerve is indicated and culminates into the retinal layer (turquoise). (c) An NdAc stained rendered eye sample where the sclera, coating the optic nerve, is clearly visible and extends into the grid-like, stiff collagenous structure. (b) and (c) were recorded at the laboratory nano-CT source. (d) An illustration of the eye structure and (e) The retinal layers of the ${\mathrm{OsO}}_{4}$ stained eye [same sample as in (b)].

Here, our previous assumptions following the qualitative assessment of the laboratory scans can be confirmed, however, now at much increased resolution and contrast. NdAc seems to significantly enhance contrast of cones and rods as well as the sclera and the Ganglion cell layer while ${\mathrm{OsO}}_{4}$ and LIS target the choroid. While cones and rods also seem to be targeted by the stains, ${\mathrm{OsO}}_{4}$ lacks any specificity and increases the attenuation in the sample uniformly, with the heavy osmium atoms causing a strong attenuation of photons in the tissue. LIS on the other hand is very favorably binding to the choroid, decreasing the number of photons passing through the sample, thus decreasing the SNR, while other layers of interest in the retina seem to only experience a minor increase in contrast. It is worth noticing that ${\mathrm{OsO}}_{4}$, which is known to strongly bind to lipids, yields strong contrast for adipose tissue and eye muscles right behind the eyeball, allowing for an easy segmentation and detailed investigation of these structures [Fig. 5(b)].

### 3.3 Quantification of Staining Results

**Fig. 6:**
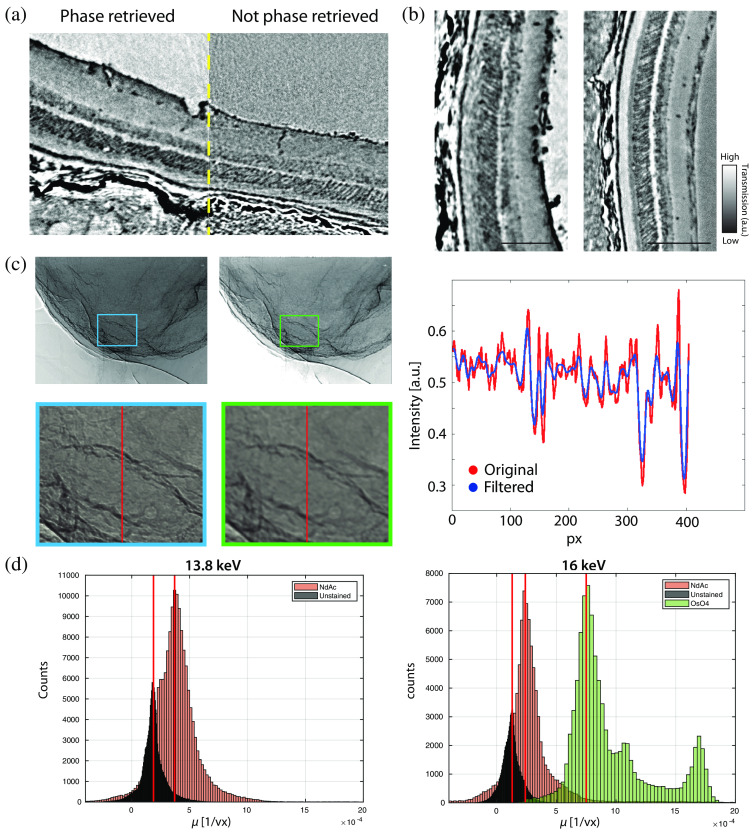
Quantitative analysis of the NdAc staining from synchrotron measurements. (a) Reconstructed slice (left) with and (right) without phase retrieval, demonstrating the benefit of phase retrieval for the image quality and quantitative contrast. The example shows NdAc stained retinal layers recorded with the synchrotron setup. (b) Qualitative comparison between NdAc stained (left) and unstained retina (right) at 13.8 keV. Scale bar: 100  μm. (c) Illustration of the workflow associated with suppressing the phase effects by local averaging of the projection (left) to create an absorption-dominated image (center). The line plot (right) shows the corresponding reduction of edge enhancement. (d) Attenuation per voxel shown for similar region of NdAc, ${\mathrm{OsO}}_{4}$, and unstained samples in histograms for 13.8 and 16 keV. The red lines mark the attenuation value where the number of counts peaks. By subtraction from the unstained peak, the attenuation of NdAc and ${\mathrm{OsO}}_{4}$ is determined.

Tomography with monochromatic SR allows to draw quantitative conclusions on the composition when modeling the absorption coefficients and/or the reconstructed phase shifts based on atomic form factors. Since the phase retrieval scheme used here yields images of coupled phase and absorption contrast based on its homogeneous object approximation, it is per se not straightforward to extract the local concentration of the stain, i.e., a spatially varying stochiometry. However, in propagation based XPCT, the contrast of the low spatial frequencies is dominated by absorption. Therefore, we can artificially generate a “pure” absorption image by local averaging the projections such that the phase effect cancels out. This is done only for the purpose of quantifying the local concentration in a coarse-grained sense, see the corresponding workflow shown in Fig. 6(c). The reconstructed absorption coefficients in the reconstruction volume are then compared between a NdAc stained sample and an unstained sample, see Fig. 6(b), scanned both at 13.8 and 16 keV. To this end, the projections are filtered, e.g., with a median filter (8×8  pixel), allowing one to take only the attenuation into consideration. A comparable region of interest involving the retinal layers with 400×400  pixel (16 keV) is then cropped from a reconstructed slice. Histograms of the gray values are shown in Fig. 6(d). By subtraction of the attenuation peaks, we obtain the additional attenuation coefficients (or more precisely the histogram thereof) attributed to NdAc in units (1/vx), allowing us to effectively isolate the staining component from the tissue and to infer its atomic density based on the known atomic form factor ($f^{\prime \prime }$ component).

The refractive index

n=1−δ+iβ (2)

shows a dependence of the dispersion term δ and the imaginary part

$$\beta =\frac{r_{0}\lambda^{2}}{2\pi }\rho_{a}f^{\prime \prime }(\omega ),$$ (3)

where $r_{0}=2.82\cdot 10^{-15}\;m$ denotes the Thomson scattering length, $\rho_{a}$ is the number density of atoms, λ is the photon’s wavelength, and $f^{\prime }(\omega )$ is the atomic scattering factor and real part of the correction derived from the Kramers–Konig relation. The linear attenuation coefficient is related to β as

$$\mu =\frac{4\pi \beta }{\lambda }$$ (4)

and is directly measured by the number of photons reaching the detector.

Substituting Eq. (4) into Eq. (3), we obtain an atomic mass density of

$$\rho_{a}=\frac{\mu }{2r_{0}\lambda f^{\prime \prime }(\omega )}.$$ (5)

**Fig. 7:**
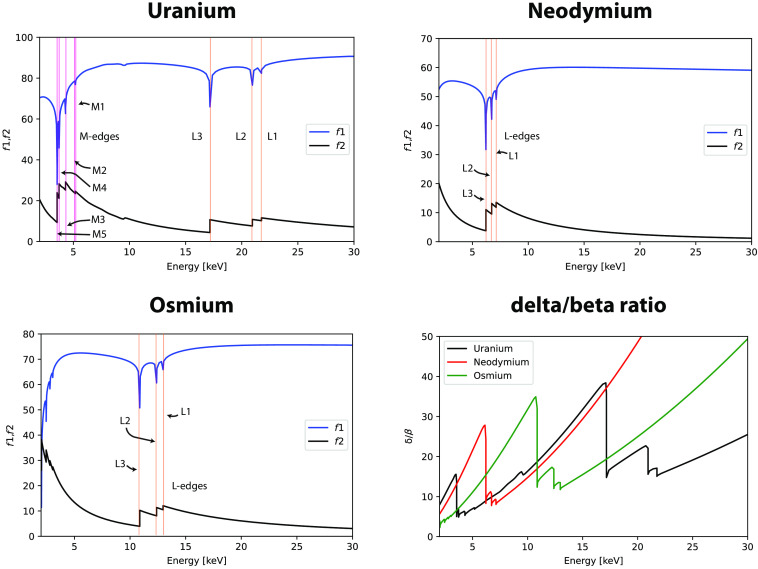
Plots of atomic scattering factors $f^{\prime }$ and $f^{\prime \prime }$ for uranium, neodymium, and osmium (with denoted M- and L-edges) and their energy-dependent δ/β ratios [Eq. (6)].

Since we know λ (λ[Å]=12.938/E[keV]), the number density and subsequently the mass density ($\rho =\rho_{a}A/N_{A}$) with the known atomic mass A and the Avogadro constant $N_{A}$ can be calculated. The atomic scattering factors $f^{\prime }$ and $f^{\prime }$ for uranium, osmium, and NdAc are plotted in Fig. 7, where the absorption edges (L, M) are indicated. To investigate their optical properties in regard to the relationship between absorption and refraction, the δ/β ratio is calculated as^47^

$$\delta /\beta =\frac{Z+f^{\prime }}{f^{\prime \prime }}.$$ (6)

From the plots, a significantly higher ratio can be inferred for neodymium at energies above 17 keV and between 2.5 and 6 keV, where therefore an advantage of neodymium can be expected for phase contrast imaging compared to uranium.

At 13.8 keV, the difference of the attenuation peaks was determined as $1.8250\cdot 10^{-4}(1/\mathrm{vox})$. Substituting this into Eq. (5), an approximate number density for NdAc of $0.0117(\mathrm{Nd}/{\mathrm{nm}}^{3})$ is obtained. To check consistency, the procedure was repeated for samples scanned at 16 keV, yielding a number density of $0.0105(\mathrm{Nd}/{\mathrm{nm}}^{3})$. In contrast, ${\mathrm{OsO}}_{4}$ shows a difference in attenuation peaks from the unstained sample of about $6.2\cdot 10^{-4}(1/\mathrm{vox})$ resulting in an estimated number density of $0.0249(\mathrm{Nd}/{\mathrm{nm}}^{3})$. Number densities and estimated free distances between atoms are listed in Table 3.

**Table 3:** Number densities and estimated mean distance between Nd atoms for different energies.

	NdAc (13.8 keV)	NdAc (16 keV)	${\mathrm{OsO}}_{4}$ (16 keV)
Number density ($\mathrm{Nd}/{\mathrm{nm}}^{3}$)	0.0117	0.0105	0.0249
Mean distance (nm)	2.73	2.83	2.13

## 4 Discussion

The results indicate NdAc to be a promising stain for XPCT, which is much better suited for phase contrast than, for example, UAc, based on the different X-ray optical properties resulting from the lower atomic number, in particular its higher δ/β ratio. At the same time, the binding properties are similar to those known for UAc, e.g., preferential binding to anionic groups such as phosphate and carboxyl groups at cell surfaces, based on the triple valency of the ${\mathrm{Nd}}^{3+}$ cation. Note that NdAc is an anionic salt with a solubility of 7.77 mol/L. From the strong non-linear electrostatics expected for a triple valency, one may expect that a particularly strong interaction may take place with anionic lipids, such as phosphatidylserine, which in neuronal cells, however, is mainly located in the cytosolic leaflet of the plasma membrane, and hence not easily accessible. It remains elusive to us why the strong contrast for the endothelium is observed, which, however, makes NdAc particularly promising for studies of the vasculature in neuronal tissue. In the murine eye, NdAc also seems to be targeting rods and cones in the retina as well as the Ganglion cell layer and in particular sclera. In comparison to ${\mathrm{OsO}}_{4}$, we observe a stronger nuance and selectivity when it comes to stain deposition and targeting of some tissue structures, providing the opportunity to study these structures in more detail without over-contrasting. Atom density estimates based on the analysis of the absorption coefficients should be further refined in subsequent work to draw conclusion about the selective partitioning into different tissue regions and the amount of stain deposited in a certain voxel. At the same time, NdAc solutions could be tested in controlled *in vitro* suspension by small-angle X-ray scattering to probe the interaction with selected lipids or biomolecular structures in quantitative terms. From the present work, we can also infer that the number density of NdAc is considerably smaller than that of ${\mathrm{OsO}}_{4}$. Importantly, its larger δ/β-values for a wide range of photon energies, making it a promising candidate stain for XPCT. Note, however, that at the resolution tested, the main advantage of the stained versus unstained samples derived from the fact that the stain opens up new imaging opportunities for laboratory instruments. Contrarily, SR already gave very convincing image quality also for unstained samples. To this end, high resolution/high magnification in the holo-tomography regime would be an interesting next step.

In summary, we could show that NdAc penetrates the sample uniformly and can increase contrast and resolution at the CNS cyto-architectural level, at least for some targets and for laboratory instruments. While there is an overall superior image quality at laboratory sources, it still remains to be clarified, how to interpret the binding and staining patterns, for example, by further correlative imaging with conventional histology. Note that the present example of murine retina in whole-eye preparations is only a very specific example, which needs to be complemented by a wider range of tissues. Due to its more favorable optical constants, NdAc is more compatible with most X-ray phase-retrieval approaches than most other EM heavy metal stains and can thus help to translate synchrotron quality to the laboratory.

### 4.1 Animal Welfare

Mice were housed in the mouse facility of the Max Planck Institute of Experimental Medicine with controlled ventilation (inward airflow, exhaust to the outside), temperature ($21\pm 2^{{}^{\circ}}C$) and controlled rel. humidity (55%±10%) in individually ventilated cages. Mice were group-housed 3 to 5 mice per cage in a 12-h dark/light cycle with ad libitum access to food and water. For the procedure of sacrificing vertebrates for subsequent preparation of tissue, all regulations given in the German animal welfare law (TierSchG §4) are followed.

## 5 Appendix: Supporting Information

Video 1. Osmium tetroxide stained whole eye sample recorded with in-house nano-CT system. (MPG, 52.6 MB [URL: https://doi.org/10.1117/1.JMI.10.5.056001.s1]).

## Code, Data, and Materials Availability

CT-reconstruction data, measurement settings and analysis scripts have been deposited in GRO.data (DOI: https://doi.org/10.25625/ZOHHEI). All study data are available upon request.
